# Experiences of maternity care: Is the perspective of health care professionals respectful care while that of women obstetric violence?: A qualitative study

**DOI:** 10.1097/MD.0000000000041467

**Published:** 2025-02-07

**Authors:** Merve Çamlibel

**Affiliations:** a Faculty of Health Sciences, Department of Obstetrics and Gynecology Nursing, Burdur Mehmet Akif Ersoy University, Burdur, Turkey.

**Keywords:** midwifery, nursing, obstetric violence, qualitative study, respectful maternity care

## Abstract

Women and health professionals may experience a number of barriers and difficulties related to maintaining and improving respectful maternity care (RMC). The aim of this study is to determine the perceptions of women who had vaginal delivery and nurses and midwives as health professionals about RMC in the care process from the perspectives of both groups. A descriptive qualitative design was used, analyzing data from semistructured interviews conducted with 11 women who had vaginal deliveries and 12 midwives and nurses. Data were analyzed using content analysis. Three main themes emerged from the content analysis: “barriers to RMC,” “RMC practices,” and “demands (expectations and suggestions) for RMC.” The study underscores the importance of health professionals and women viewing each other as collaborators. However, there should be a conducive environment that motivates healthcare professionals and provides opportunities for professional development and evidence-based practice. Government and hospital management should acknowledge these barriers and support efforts to empower both women and their caregivers (midwives, nurses, or physicians) to address them. Furthermore, the research results could aid in formulating national and international clinical recommendations designed to prevent obstetric violence.

## 1. Introduction

Maternity care encompasses a range of healthcare services delivered by physicians, nurses, midwives, hospitals, or birthing centers to pregnant women throughout pregnancy (prenatal care), childbirth, and the postpartum period (postnatal care).^[1]^ Respectful maternity care (RMC) is highlighted by the World Health Organization as an essential concept for women’s rights and safety. RMC refers to the provision of humane and dignified treatment to a woman.^[2]^ On the other hand, the quality of maternity care is reduced in disrespectful and abusive behaviors and environments. Women’s experience of the care process is an important health indicator for both women and health professionals.^[3]^ Women’s perceptions of the pregnancy and childbirth experience are known to be affected by 4 main factors, including personal expectations, support from caregivers, quality of the relationship between caregiver and care recipient, and participation in the decision-making process.^[4]^ During their maternity care process, women interact and communicate mostly with nurses and midwives among health professionals.^[5]^ Therefore, the perceptions, experiences, and emotions of health professionals are an important factor in the provision of women’s psychosocial support needs and the positive or negative perception of the process.^[6]^

In most studies, positive communication skills and respect have been reported to be women’s greatest expectations from health professionals. These respectful care behaviors needed by women have been proposed and recommended by the World Health Organization.^[7,8]^ Health professionals’ caring philosophy, values, and characteristics are important components for maternity care quality and maternity-friendly care delivery.^[9]^

The concept of a mother-friendly hospital, developed by the Coalition for Improving Maternity Services in 1996, includes 12 steps to be followed while providing care to the mother and the baby.^[10]^ The principle of respect, dignity, and informed choice is the primary one. In Turkey, 98% of births occur in hospitals, with 18% of public hospitals holding the designation of “mother-friendly hospitals.” Both midwives and nurses are actively involved in prenatal and postnatal care. The delivery process is primarily overseen by physicians or midwives. Low-risk pregnancies are typically managed by midwives, while obstetricians intervene in cases of labor complications or difficult deliveries.^[11]^ However, these metrics do not fully capture women’s perceptions of care, especially their experiences with communication, respect, and emotional support, thus failing to comprehensively reflect care quality.^[12]^ Furthermore, health workers’ personal and professional attitudes also influence the care process, alongside their education and exposure to different maternity care models.^[13]^

Receiving the views of women and health professionals is important when maternal health programs are designed, implemented, monitored, and evaluated. Several studies in the literature have investigated midwives’ and women’s RMC-related experiences.^[14,15]^ However, no qualitative studies worldwide were found to have synthesized the perceptions and experiences of women and nurses/midwives as health professionals about RMC by including both groups in tandem.

The purpose of this study is to determine the perceptions of women who had vaginal delivery and nurses and midwives as health professionals about RMC in the care process from the perspectives of both groups.

## 2. Materials and methods

### 2.1. Design

This study used a qualitative descriptive approach guided by Husserlian phenomenology, as developed by Husserl.^[16]^ Husserl framework emphasizes that knowledge of experiences is derived from within the experience itself. This method involves describing a phenomenon (an event or experience) from the perspective of the individuals involved. The rationale for adopting Husserlian phenomenology was to gain deeper insights into the unique experiences of each woman and healthcare professional (including midwives and nurses) within their specific contexts. This research method was deemed most suitable for exploring the perceptions and experiences related to RMC among women and healthcare providers. The study adhered to the Consolidated Criteria for Reporting Qualitative Research guidelines during its preparation.^[17]^

### 2.2. Participants

The snowball sampling method was utilized to access the participants between February and November 2023. Snowball sampling (or chain sampling) is a nonprobability sampling technique that allows existing study subjects to recruit future subjects among their acquaintances.^[18]^ The study was announced on social media accounts primarily followed by postpartum women and midwives. The participants of the study were recruited through platforms such as Facebook and Instagram particularly. Women and health professionals who volunteered to participate read the online informed consent form and ticked the box indicating the option “I agree to participate in the study.” After the first participant was contacted via social media, other women and midwives were subsequently reached in a chain-like manner until data saturation was achieved. In line with the literature, the interviews were conducted until no new information was obtained.^[18,19]^ The study was completed with 11 women who had vaginal delivery and 12 health professionals.

The inclusion criteria for women are given as follows: volunteering to participate in the study, having a vaginal delivery at 37 weeks of gestation or above in a mother-friendly state hospital, being over 18 years of age, being able to speak and understand Turkish, not having any psychiatric disorders, having a healthy pregnancy, and not having any chronic diseases. Inclusion criteria for health personnel (nurses and midwives) are given as follows: volunteering to participate in the study and having worked in the intrapartum/postpartum unit of a mother-friendly state hospital for at least 6 months.

### 2.3. Data collection

In line with the purpose of the study, qualitative interviews were conducted in 2 different groups (health personnel and women who had vaginal delivery). Data were collected through the Personal Information Form and a semistructured interview form.

#### 2.3.1. Personal information form for women

The Personal Information Form for women consisted of questions related to women’s sociodemographic and obstetric characteristics (age, education level, perceived economic level, employment status, number of pregnancies, gestational week at the time of delivery, etc).

#### 2.3.2. Personal information form for health professionals

The Personal Information Form for health professionals consists of questions about health personnel’s sociodemographic characteristics such as years of employment, education level, marital status, and age.

#### 2.3.3. Semistructured interview form

The semistructured interview form consisted of the following questions that aimed to obtain data about women’s RMC experiences: how was your childbirth and postpartum experience? What did you experience after giving birth, how did you feel? Can you explain? Can you explain your experience of RMC services? What do you think RMC services should include? What are your suggestions for RMC services? and What were your experiences with the attitudes and approaches of health personnel in the care process after your admission to the hospital?^[12,20]^

The semistructured interview form consisted of the following questions aiming to learn about the experiences of health personnel’s RMC: What do you think about RMC and its components? Can you tell us about your clinical experiences concerning RMC? As health personnel, what are the facilitating factors in the provision of RMC services to your patients? As health personnel, what do you think are the barriers to the provision of RMC services? and As health personnel, how can you improve RMC services in hospitals? and what are your suggestions?

All the interviews continued with basic questions, and additional questions (what do you mean, can you explain more, etc) were asked according to the interviewee’s responses. All the interviews were conducted by the author, who holds a PhD degree. The author attended courses on qualitative research methodology and has research on RMC and qualitative research methodology. Interviews with the participants were conducted via cell phone (WhatsApp, voice calls, etc) at the time determined by the participants, and the interviews were audio recorded. The interviewees and the interviewer had no relationships. The audio recordings were transcribed verbatim by the researcher and sent to each woman to check for any missing parts. All the participating women confirmed the statements in the texts. Data were analyzed after the women’s consent was received. Interviews with women ranged from 20 to 51 minutes, while interviews with health personnel ranged from 16 to 54 minutes.

### 2.4. Ethical considerations

Ethics approval (decision number 2022/946 dated December 7, 2022) was obtained from the Burdur Mehmet Akif Ersoy University Non-Interventional Clinical Research Ethics Committee. The study adhered to the principles outlined in the Declaration of Helsinki. Each participant, both women and health personnel, reviewed the informed consent form and indicated their agreement by checking the box labeled “I agree to participate in the study.” Verbal consent from participants was obtained after starting the audio recordings. Voice recordings and transcriptions were stored in computer files protected by passwords.

### 2.5. Data analysis

Descriptive statistics were utilized to analyze sociodemographic data obtained from the women and health personnel. Transcribed interview data were analyzed using content analysis as described by Graneheim and Lundman (2004). The analysis was conducted independently by 2 researchers who were not involved in the study. Two independent faculty members were consulted concerning the compatibility of the codes, and in the case of different opinions, the coders tried to achieve consensus and compatibility. Each content was summarized, and each unit of meaning was assigned a code. The codes were then analyzed in terms of the aims of the study, and conceptually similar codes were categorized. Themes were identified and named after the categorization process. The findings and quotations obtained from the participants are presented without making any changes.^[21]^ The participants were coded as W (women), M (midwife), and N (nurse) to protect the privacy and the confidentiality of their identity information. In the findings section, the codes are given in parentheses next to the participants’ quotes.

### 2.6. Rigor

The validity and reliability of this study were ensured using the principles of credibility, transferability, consistency, and confirmability.^[22]^ The interviews with the participants lasted from 16 to 54 minutes to ensure long-term interactions for internal validity. The interviews were ended when appropriate and necessary. Two faculty members who were not involved in the study reviewed the transcripts individually to ensure that the interviewer was not influenced by the data obtained from the participants. Different and common points were then reviewed for each group. The study phases were carried out according to the Consolidated Criteria for Reporting Qualitative Research checklist. External validity was ensured through the snowball sampling method, chosen for its appropriateness in this study. Researchers presented the data based on emerging themes, maintaining a thick description approach without adding comments. For internal reliability, consistency in the treatment of interviewees was maintained across all stages of the study, with uniform use of the same voice recorder and interview form. Regarding external reliability, an expert in the field reviewed all data collection tools, voice recordings, raw data, codes, and themes, ensuring their integrity and readiness for future use.^[23]^

## 3. Results

The mean age of the women is 29.27 ± 3.37 years. Three of the women have a primary school degree, and their mean gestational age at birth is 38.81 ± 1.25 weeks, with a minimum of 1 and a maximum of 2 parities. There are 6 midwives and 6 nurses among the health professionals, with a mean age of 33.91 ± 7.79 years. Their mean professional experience is 10 years (Tables 1 and 2).

**Table 1:** Descriptive characteristics of women (N = 11).

ID code (W)	Age, yr	Education level	Employment status	Parity	Gestational age at birth, wk
W1	29	University	Employed	1	39
W2	24	High school	Unemployed	1	37
W3	34	University	Unemployed	2	38
W4	26	University	Unemployed	1	39
W5	28	High school	Employed	1	40
W6	32	Primary school	Unemployed	2	37
W7	34	University	Employed	2	38
W8	29	University	Employed	1	41
W9	25	Primary school	Unemployed	1	39
W10	30	Primary school	Employed	2	39
W11	31	High school	Employed	2	40

W = women.

**Table 2:** The descriptive characteristics of healthcare professionals (N = 12).

ID code (M and N)	Age, yr	Education level	Marital status	Duration of professional experience, yr
M1	27	University	Married	4
M2	26	University	Single	3
M3	32	High school	Divorced	10
M4	40	High school	Married	15
M5	30	High school	Single	8
M6	49	University	Married	23
N1	28	University	Single	5
N2	36	University	Married	11
N3	39	High school	Married	15
N4	29	High school	Single	5
N5	26	University	Married	4
N6	45	University	Married	18

M = midwife, N = nurse.

Content analysis revealed 3 main themes: barriers to RMC, RMC practices, and demands (expectations and suggestions) for RMC. Subthemes were also included under the 3 themes determined in the study (Table 3).

**Table 3:** Issues concerning RMC from the perspectives of women and health care professionals.

Themes	Subthemes
Barriers to RMC	Barriers related to women Barriers related to partner/family caregivers Barriers related to health professionals Barriers related to the healthcare system
RMC practices	Supportive practices Improvisational practices
Demands (expectations and suggestions) for RMC	Professional experience and professional approach Individualized women-centered care

RMC = respectful maternity care.

### 3.1. Theme 1: barriers to RMC

Responses given by the women and health professionals included barriers related to women, barriers related to partner/family caregivers, barriers related to health professionals, and barriers related to the healthcare system.

#### 3.1.1. Subtheme 1: barriers related to women

Participating health personnel stated that women had different attitudes toward childbirth. They emphasized that some women were more dependent, while others were more independent.

“Finding a middle ground is very difficult in some women, which makes the process disrespectful and communication problematic.” (N3)

“We want women to be active; we give them freedom of movement, but the woman does not even want to stand up; she wants us to perform her birth from the bed she is in.” (M6)

The health personnel stated that due to their lack of knowledge, women could not understand what was explained in the process and, therefore, could not be involved in the process and left all responsibility to the health personnel.

“I give information and explanations so that women can manage the process themselves, but some of them do not listen to me and try to do what they heard from their environment. Hence, they can neither give birth nor breastfeed. They ask for our support in the whole process.” (N4)

“I don’t think abuse cases are experienced; it’s just that sometimes women want too much attention. When they don’t get this, they think they are neglected and left alone.” (M3)

#### 3.1.2. Subtheme 2: barriers related to partner/family caregivers

Health professionals emphasized that they had communication problems, especially with women’s partners or people who were with the woman at the time of labor or in the postpartum period, which negatively affected the care process.

“The patient’s relatives shout that the woman is in pain, why don’t you do something? When we involve them too much in the process, we have such problems.” (M4)

“They expect us to be always ready to provide service. After the delivery, they want us to be with them all the time about things related to the baby.” (N6)

#### 3.1.3. Subtheme 3: barriers related to health professionals

Participating women mentioned health professionals’ varying behaviors based on individual factors.

“The process was long because it was my first childbirth. The nurses and midwives had a very bad approach to me at night; they did not pay attention, but the ones who came after the shift change were good.” (W1)

Some health professionals noted differences in the work and care perceptions among their colleagues.

“While some of my friends perceive work as a burden, some others feel the opposite.” (M5)

“Some of my friends do not care about women’s opinions. I think sometimes we do not listen to women and just say ‘I know the process; I think what’s best for you.’” (M4)

Participating women reported that some health professionals shouted at them, especially during the second stage of labor.

“I think I didn’t push properly at the right time, and the midwife shouted. But I do not think that it was deliberate; they were trying to do their job.” (W9)

#### 3.1.4. Subtheme 4: barriers related to the healthcare system

Participating health personnel reported a lack of motivation due to the healthcare system.

“We midwives manage the whole process. Physicians are not involved in the process, but the childbirth fee is paid to them. It would be better if we were at least financially improved.” (M1)

Regarding the healthcare system, participating women stated that private rooms were present, but being in a crowded environment at the time of delivery affected privacy negatively.

“I was in a private room during labor, but I think there were 7-8 people in the room at the time of delivery. I felt like an experimental animal. I have no idea what was done. I am here now, my baby is healthy, and that’s what matters.” (W5)

Participating women stated that they wanted the physicians who followed them during pregnancy to be present at the time of delivery.

“I wanted my doctor who took care of me during the whole pregnancy process, but he did not come. I did not know that midwives were supposed to perform the delivery. My doctor came at the last moment and he did not do anything.” (W4)

### 3.2. Theme 2: RMC practices

Both health professionals and some women in our study stated that they were provided with RMC practices in the clinic.

#### 3.2.1. Subtheme 1: supportive practices

Participating women mentioned health professionals’ supportive and motivating approaches.

“The nurses and midwives were very supportive. I felt that they were with me; they held my hand. They guided me on what to do both during and after the delivery.” (W7)

#### 3.2.2. Subtheme 2: improvisational practices

Both women and health personnel in the study stated that some practices developed spontaneously.

“They pressed on my abdomen; they cut from below; they induced labor… Actually, many things happened, but they didn’t ask for my consent. I know that all of these things were done for me so that I could hold my healthy baby.” (W11)

“Consent sometimes happens at the moment; sometimes you see that the woman is not in a condition to listen to it, so it develops improvisationally.” (M2)

### 3.3. Theme 3: demands (expectations and suggestions) for RMC

The “demands (expectations and suggestions) for RMC” theme included the subthemes of professional experience, professional approach, and individualized women-centered care. Women and health professionals participating in the study identified factors that facilitate their ability to provide improved RMC.

#### 3.3.1. Subtheme 1: professional experience and professional approach

The provision of a professional approach to women was the health professionals’ demand for RMC practices.

“Actually, there should be turnover among the staff. The same people should not be in the delivery room for years. Staff can have a rotation between the ward and the delivery room for fresh perspectives and motivation.” (M6)

“If we show that we are professionally experienced in our work and if we can empathize, there are actually no problems. Then they trust us.” (M2)

“It is important to be able to assure women that they are free in their choices and they will not be subjected to mistreatment.” (M1)

#### 3.3.2. Subtheme 2: individualized women-centered care

Both women and health personnel indicated that they were aware of the importance of individualized woman-centered care for maintaining RMC.

“Care should start between the midwife and the pregnant woman before the delivery process. Otherwise, the woman does not recognize and cannot trust the midwife; her eyes are looking for her doctor.” (M5)

“I know that I am supposed to lead the process, yet one still wants to receive support, a positive word, and encouragement and then to be empowered.” (W8)

## 4. Discussion

This study aimed to determine the perceptions of women who had vaginal delivery and nurses and midwives as health professionals about RMC in the care process.

Participating health personnel and women identified a range of barriers to RMC in this study. Barriers to RMC included barriers related to health professionals, barriers related to women, barriers related to women’s partners and family caregivers, and barriers related to the healthcare system. Health personnel play an important role in helping women cope with barriers to RMC.^[24]^ However, health personnel in this study also emphasized barriers associated with health policies, women, or their family caregivers. Participating women stated that individual differences in attitudes and expectations between health personnel and patient relatives were reflected in RMC practices. Individual differences were also reported to be experienced in other studies, which are related to individuals’ professional ethics and morality.^[14]^ In addition, training for health personnel on RMC was found to effectively improve care behaviors.^[7]^ Therefore, it is important to implement programs to strengthen the provision of RMC and to provide educational activities considering the health personnel’s values, professional ethics, and needs.

This study also found that having a healthy baby was the most important thing for women because, although there were problems related to lack of consent, lack of information, and privacy in some practices that develop spontaneously during labor, women reportedly did not care about this situation. The results of the study also showed that many women did not know about or were not aware of obstetric violence and perceived violence as part of the childbirth process.^[25]^ Similar to our study results, women accept procedures without questioning, hesitate to express their demands, and focus only on having their babies healthy without even knowing that their rights are being violated. In their study conducted in Turkey, Aşci and Bal^[26]^ reported that ≈3 of 4 women reported being exposed to obstetric violence during labor. In addition, similar to our study findings, other studies also reported that although health personnel were aware that informing women was related to RMC, sometimes, differences were experienced in practice.^[15,27]^ In some studies, health personnel reported that they focused more on delivering a healthy and live baby than thinking about the abuse of women.^[28]^

The women also reported that while, in a mother-friendly hospital, they expected their delivery to be performed by the physician who followed them during pregnancy, they experienced a lack of trust in deliveries performed by midwives and barriers to privacy associated with the healthcare system because several others were observing their delivery. A woman’s relationship with health professionals before, during, and after pregnancy and delivery is vital in terms of positive maternity care outcomes. Maintaining the trust relationship established between women and health professionals starting from the antenatal period in the labor and postpartum processes as well is important in terms of RMC practices.^[29]^ While health personnel emphasize the importance of including women in the process, talking about and discussing expectations for both women and health personnel in formal training to be provided to women starting from the antenatal period is an important factor in terms of women’s readiness for the delivery and postpartum process.^[7]^

Women in this study also reported feeling supported, motivated, and empowered when they were provided with RMC practices. The women stated that they felt like human beings again when midwives were friendly to them and made them feel valued.^[15,30]^ In this study, both health professionals and women were aware of the importance of RMC, yet they mentioned the importance of professional experience, empathic approach, and professional approach and the need to provide individualized woman-centered care specific to each woman to overcome the obstacles that they experienced. Women in labor need to be able to establish a trusting connection with the midwives and obstetricians who provide care for them. The concept of a positive childbirth experience consists of important components such as “woman-centered care, feeling supported, feeling safe, feeling in control, and feeling respected.”^[6]^

### 4.1. Limitations

This study has limitations. As the participants were reached online, only those who had an internet connection and used Instagram, Facebook, or WhatsApp were recruited for the study. In this way, many different units with the title of mother-friendly hospital were reached, and diversity was achieved among the participants. However, the results of the study cannot be generalized to all women and health professionals. The results of this study are limited to the participants in the sample in Turkey because each country’s maternity care protocols may be different. These limitations should be considered when interpreting the findings. Despite these limitations, this qualitative study has various strengths. It is the first qualitative study to evaluate both sample groups together in order to synthesize the perceptions and experiences of women and nurses/midwives as health professionals regarding RMC.

## 5. Conclusion

This study found that there are barriers to maintaining RMC for both women and health professionals. However, some of these factors were reported to be external problems stemming from the healthcare system. Some of the barriers were found to be internal problems (philosophy of care, lack of knowledge, etc) arising from both health professionals and women. All mutual factors that form a whole were actually expressed as barriers. Health professionals and women who are collaborators for RMC continue to face various challenges in the implementation of RMC. Both women and health professionals agree that RMC has a significant impact on the quality of care. Therefore, health personnel’s skills and knowledge on RMC should be kept up to date, and working conditions and motivational resources should be provided to facilitate women-centered care. As for women, increasing their readiness both in the antenatal and postnatal period with training starting from the antenatal period is considered to make a significant contribution to maintaining mutual RMC.

This article may enable health professionals to recognize facilitators and barriers to the provision of RMC services to women. Health professionals can plan interventions to eliminate barriers and integrate RMC starting from the antenatal period to maintain facilitating factors. Further descriptive and experimental studies are needed to assess specific issues related to RMC in the clinical field.

## Acknowledgments

The authors would like to thank the participants for their time and efforts in this study.

## Author contributions

**Conceptualization:** Merve Çamlibel.

**Data curation:** Merve Çamlibel.

**Formal analysis:** Merve Çamlibel.

**Investigation:** Merve Çamlibel.

**Methodology:** Merve Çamlibel.

**Resources:** Merve Çamlibel.

**Software:** Merve Çamlibel.

**Supervision:** Merve Çamlibel.

**Validation:** Merve Çamlibel.

**Visualization:** Merve Çamlibel.

**Writing – original draft:** Merve Çamlibel.

**Writing – review & editing:** Merve Çamlibel.
